# Sulforaphane Regulates eNOS Activation and NO Production via Src-Mediated PI3K/Akt Signaling in Human Endothelial EA.hy926 Cells

**DOI:** 10.3390/molecules27175422

**Published:** 2022-08-24

**Authors:** Ying Zhang, Pham Ngoc Khoi, Bangrong Cai, Dhiraj Kumar Sah, Young-Do Jung

**Affiliations:** 1 Department of Cell Biology, School of Medicine, Jiangsu University, Zhenjiang 212013, China; 2 Research Institute of Biomedical Sciences, Chonnam National University Medical School, Gwangju 501-190, Korea; 3 Faculty of Basic Medical Sciences, Pham Ngoc Thach University of Medicine, Ho Chi Minh City 740500, Vietnam; 4 School of Pharmacy, Henan University of Chinese Medicine, Zhengzhou 450046, China

**Keywords:** sulforaphane, Src, Akt, eNOS, NO, EA.hy926

## Abstract

Sulforaphane (SFN) is a naturally occurring isothiocyanate that is abundant in many cruciferous vegetables, such as broccoli and cauliflower, and it has been observed to exert numerous biological activities. In the present study, we investigate the effect of SFN on eNOS, a key regulatory enzyme of vascular homeostasis and underlying intracellular pathways, in human endothelial EA.hy926 cells. The results indicate that SFN treatment significantly increases NO production and eNOS phosphorylation in a time- and dose-dependent fashion and also augments Akt phosphorylation in a time- and dose-dependent manner. Meanwhile, pretreatment with LY294002 (a specific PI3K inhibitor) suppresses the phosphorylation of eNOS and NO production. Furthermore, SFN time- and dose-dependently induces the phosphorylation of Src kinase, a further upstream regulator of PI3K, while PP2 pretreatment (a specific Src inhibitor) eliminates the increase in phosphorylated Akt, eNOS and the production of NO derived from eNOS. Overall, the present study uncovers a novel effect of SFN to stimulate eNOS activity in EA.hy926 cells by regulating NO bioavailability. These findings provide clear evidence that SFN regulates eNOS activity and NO bioavailability, suggesting a promising therapeutic candidate to prevent endothelial dysfunction, atherosclerosis and other cardiovascular diseases.

## 1. Introduction

The endothelium is of vital importance to maintain vascular homeostasis, and ab-normal homeostatic functions of the endothelium contribute to the development of cardiovascular diseases such as thrombogenesis, atherosclerosis, and myocardial infarction [1,2,3,4,5]. The presence of nitric oxide (NO) is the most important factor to maintain vascular homeostasis. NO is generated from the precursor L-arginine in the catalysis of the enzyme endothelial NO synthase (eNOS) [6,7], and it regulates systemic blood pressure, vascular remodeling and angiogenesis [8]. Substantial evidence has shown that the reduced bioavailability of NO that is frequently observed in early vascular diseases is largely a result of eNOS uncoupling with reduced expression, phosphorylation, or impaired activity [9,10]. The activity of eNOS is mainly regulated by multisite phosphorylation, and the most thoroughly studied sites are the activation site at Ser 1177 and inhibitory site at Thr 495 [11].

The phosphorylation of eNOS lies downstream of Akt, which is activated by phosphoinositide-3 kinase (PI3K) via recruitment to the plasma membrane in which Akt is always phosphorylated at Ser 473 and Thr 308 [12]. Additionally, Akt has been reported to be phosphorylated on Tyr 315 and Tyr 326 by an Src kinase that is characterized as a critical upstream modulator [13]. This tyrosine kinase phosphorylation is considered to be crucial for the full activation of Akt, but is independent of the phosphorylation of serine/threonine [14]. The constitutive activation of the Src-mediated PI3K/Akt pathway has been found to promote eNOS phosphorylation at Ser 1177, restoring the NO production that exerts the endothelium-dependent vasorelaxation [15,16,17].

Pharmacological interventions that prevent eNOS uncoupling and thereby reverse endothelial dysfunction to improve cardiovascular disease have attracted significant interest. Preclinical studies have demonstrated that many drugs, such as aliskiren, eplerenone, statins nebivolol and pentaerythrityl tetranitrate, prevent eNOS uncoupling. Additionally, bioactive molecules including resveratrol, BH4, sepiapterin, folate and AVE3085 have been reported to recouple eNOS activity and reverse endothelial dysfunction [18].

Sulforaphane (SFN) is a naturally occurring isothiocyanate that is abundant in many cruciferous vegetables, such as broccoli and cauliflower, and it exerts numerous biological activities, including anticancer [19,20] and neuroprotective effects [21]. Previous reports have shown that SFN impairs endothelial functions induced by various stimuli and prevents atherosclerotic plaque growth and cardiovascular diseases [22]. However, the molecular mechanisms are not clear. In the present study, we show that sulforaphane regulates eNOS activation and NO production via Src-mediated PI3K/Akt signaling in human endothelial EA.hy926 cells.

## 2. Results

### 2.1. Effect of SFN on the Cell Viability and NO Production in EA.hy926 Cells

**Figure 1:**
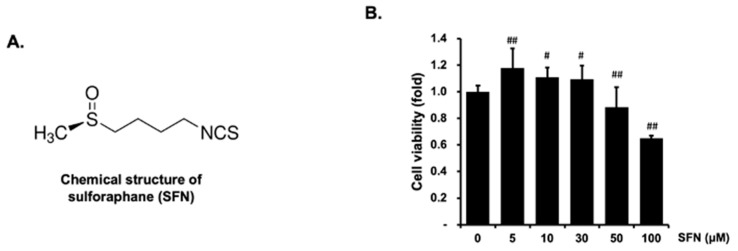
Chemical structure of SFN (**A**) and its effect on cell viability in human endothelial EA.hy926 cells (**B**). Cells were treated with the indicated concentrations of SFN for 24 h and then incubated with 10 μL MTT solution for 2 h. Each experiment was performed in triplicate, and three independent experiments were conducted. The data are presented as means ± SEM. # *p* < 0.05; ## *p* < 0.01 versus control.

An MTT assay was first used to study whether SFN at a treatment concentration ranging from 0 to100 μM caused cell damage. As shown in Figure 1B, SFN is nontoxic at a concentration below 50 μM. Thus, the treatment concentration of SFN was used from 0 to 50 μM in the following study.

**Figure 2:**
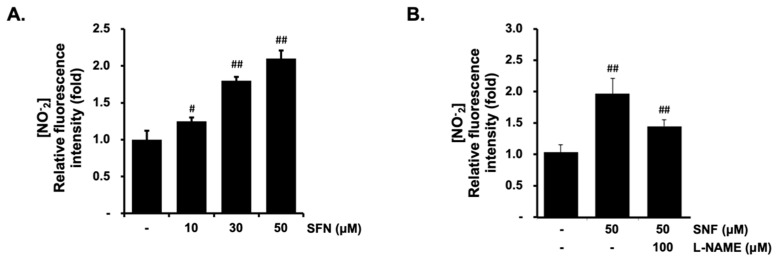
SFN increases NO production in EA.hy926 cells. Cells with 95% confluence in the chamber slides were grown and starved overnight. The cells were treated with DAF-2 or DAF-2DA with a final concentration of 1 μM for 30 min at 37 °C, rinsed three times with DMEM, and incubated in the dark, followed by treatment of SFN at the indicated concentrations without (**A**) or with (**B**) L-NAME (100 μM), which was added 30 min ahead of DAF-2DA treatment. The samples continued to be incubated for 24 h. NO production was determined by spectrofluorometer at peak excitation and emission wavelengths of 495 nm and 515 nm, respectively. The data represent the mean ± standard deviation from triplicate measurements. # *p* < 0.05; ## *p* < 0.01 versus control.

Nitric oxide (NO) production was then determined using NO-specific fluorescent dyes DAF-2 and DAF-2DA. As depicted in Figure 2A, SFN significantly augmented the production of NO in a dose-dependent manner, with the maximum response observed at 50 mM treatment, attenuated by a NO synthase-specific inhibitor L-NAME treatment (Figure 2B).

### 2.2. Effect of SFN on the Phosphorylation of eNOS in EA.hy926 Cells

**Figure 3:**
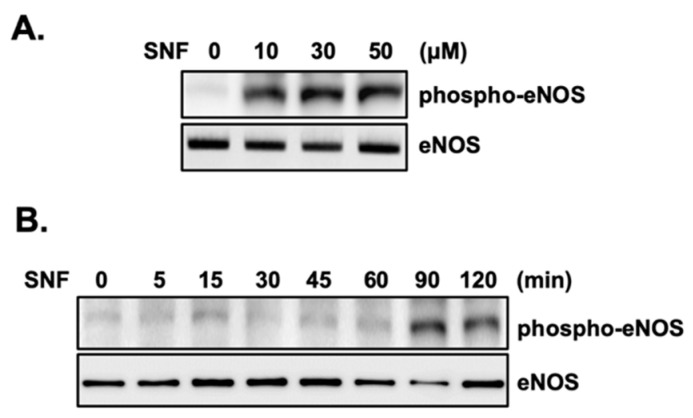
SFN increases the phosphorylation of eNOS in a concentration- and time-dependent fashion in EA.hy926 cells. The cells were treated with various concentrations of SFN for 1 h (**A**) or 50 μM SFN for the indicated time (**B**). Cell lysates were examined by Western blot using antibodies to eNOS or phosphorylated eNOS. The blots are representative of three independent assays.

To clarify the mechanism by which SFN increased NO release from EA.hy926 cells, the phosphorylation level of the endothelial nitric oxide synthase (eNOS), the active form of eNOS that is the primary physiological source of NO, was investigated. SFN exposure for 1 h increased the expression level of phosphorylated eNOS in EA.hy926 cells in a dose-dependent manner, whereas it had no effect on eNOS expression (Figure 3A). Next, we examined the effect of SFN (50 μM) on eNOS phosphorylation at different time points. The results showed that the phosphorylation level of eNOS increased when exposed to SFN with a prolonged treatment time from 5 to 120 min, with a maximum response observed at 90 min post-initiation of incubation (Figure 3B). However, there was no significant effect on the total form of eNOS, indicating that the augmentation of NO was closely associated with the increased phosphorylation of eNOS by SFN stimulation.

### 2.3. Involvement of PI3K/Akt in SFN-Induced eNOS Phosphorylation

**Figure 4:**
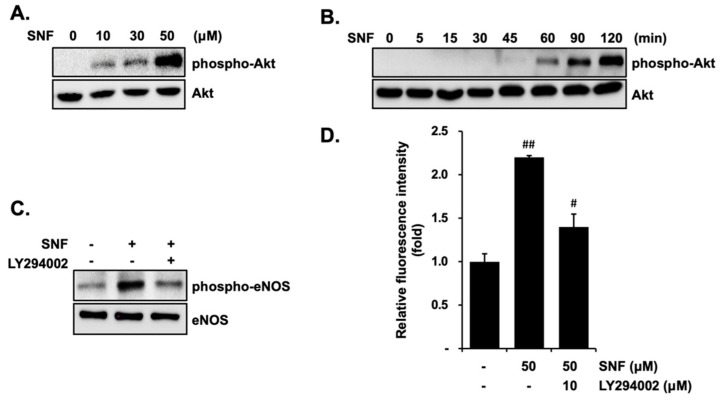
SFN stimulates the phosphorylation of eNOS via PI3K/Akt signaling in EA.hy926 cells. The cells were treated with various concentrations of SFN for 1 h (**A**) or 50 μM SFN for the indicated time points (**B**). Cell lysates were determined by Western blot using primary antibodies to Akt or phosphorylated Akt. Cells were pretreated with 10 μM LY294002 prior to treatment with or without 50 μM SFN. Cell lysates were examined by Western blot using primary antibodies to eNOS or phosphorylated eNOS (**C**), and NO production was quantified by NO measurement kit (**D**). The blots are representative of three independent assays. # *p* < 0.05; ## *p* < 0.01 versus control.

The upstream regulatory factor of phosphorylated Akt was determined to further demonstrate the mechanism involved in eNOS phosphorylation by SFN exposure in EA.hy926 cells. SFN remarkably increased Akt phosphorylation in a dose-dependent manner and reached a maximum response at 50 μM (Figure 4A). However, there was no obvious alteration in the total form of Akt. SFN-induced Akt phosphorylation had no remarkable influence when the treatment time was less than 45 min, while it showed increased expression with a time extension and reached a maximum response at 120 min post-initiation of incubation (Figure 4B). Similarly, the expression level of the total Akt did not vary after SFN treatment at different time points.

To examine the specific role of Akt in SFN-induced eNOS phosphorylation, cells were pretreated with 10 μM LY294002 (PI3K inhibitor) prior to SFN exposure. As shown in Figure 4C, SFN treatment alone significantly augmented the phosphorylated eNOS, whereas LY294002 obviously compromised the SFN-induced eNOS phosphorylation. Consistent with reduced eNOS activity, a decrease in NO production induced by SFN was also observed in the pretreatment of LY294002 (Figure 4D). Taken together, these results suggest that SFN phosphorylates eNOS and stimulates NO production through PI3K-mediated Akt activation.

### 2.4. Involvement of Src Kinases in SFN-Induced eNOS Phosphorylation

**Figure 5:**
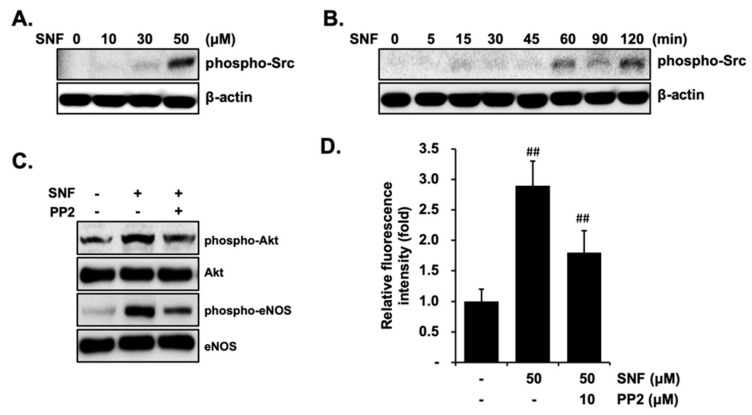
Src kinases are involved in SFN-induced eNOS phosphorylation via PI3K/Akt activation in EA.hy926 cells. Cells were treated with various concentrations of SFN for 1 h (**A**) or 50 μM SFN for the indicated time points (**B**). The cell lysates were determined via Western blot using primary antibodies to Src, phosphorylated Src or actin. Cells were pretreated with 10 μM PP2 prior to treatment with or without 50 μM SFN. Cell lysates were examined by Western blot using primary antibodies to phosphorylated Akt, Akt, phosphorylated eNOS or eNOS (**C**), NO production was quantified using an NO measurement kit (**D**). The blots are representative of three independent assays. ## *p* < 0.01 versus control.

The Src kinase family serves as a further upstream stimulator of the PI3K/Akt pathway, and it has been demonstrated that the phosphorylation of eNOS is regulated by numerous factors via Src kinase. To address the influence of the Src kinase as an activator of the PI3K underlying SFN-mediated activation of eNOS, the effect of SFN on the phosphorylation level of Src was investigated in EA.hy926 cells. As shown in Figure 5A, the phosphorylation level of Src was boosted significantly in a dose-dependent fashion and a maximum response was seen with the 50 μM SFN treatment, whereas there was no statistically significant alteration in the total form of Src. In addition, SFN time-selectively altered the level of phosphorylated Src. This had no remarkable influence on phosphorylated Src when the SFN exposure time was less than 45 min, while a significant increase was observed in phosphorylated Src expression after 60 min (Figure 5B). Next, we evaluated the effect of PP2, a specific Src kinase inhibitor, on SFN-stimulated phosphorylated eNOS and phosphorylated Akt. As shown in Figure 5C, PP2 inhibited both phosphorylated eNOS and phosphorylated Akt expression. In line with the inactivation of eNOS, PP2 also quenched SFN-induced NO production. These results indicate that SFN stimulates Src kinase, which in turn activates the PI3K/Akt pathway, ultimately leading to an increased phosphorylation of eNOS and NO production.

## 3. Discussion

**Figure 6:**
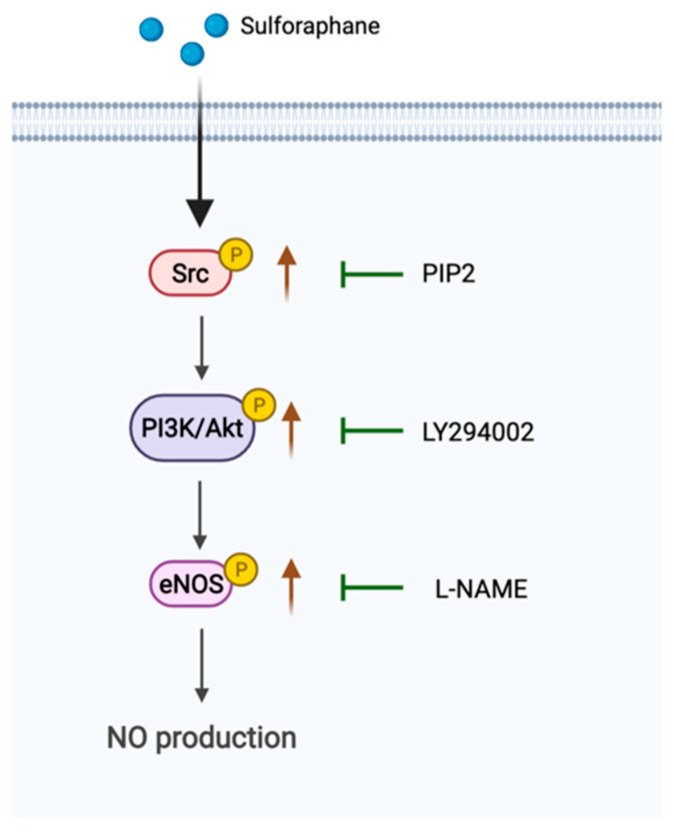
Proposed mechanism of action mediating sulforaphane-stimulated eNOS activation and NO production. SFN stimulates the activation of Src kinase that causes PI3 kinase activation, followed by Akt activation, which is an upstream kinase of eNOS and regulates eNOS activity by phosphorylation at Ser1177, ultimately leading to NO production.

In the present study, for the first time, we investigated the mechanism of action by which SFN augments eNOS activity. One of the key findings was that SFN enhances eNOS activity by phosphorylation at Ser 1177 without changing the total eNOS level, resulting in an increase in NO liberation in human endothelial EA.hy926 cells. Furthermore, SFN stimulates eNOS phosphorylation via the c-Src kinase-mediated PI3K/Akt signaling pathway, which plays a crucial role in SFN-induced eNOS phosphorylation (Figure 6).

NO is consecutively produced by eNOS to maintain vascular homeostasis by improving endothelial repair, systemic blood pressure, vascular remodeling and postnatal neovascularization under physiological conditions [5]. Substantial evidence has reported that endothelial dysfunction is closely associated with the reduced activity of eNOS, resulting in a reduction in NO production and increased oxidative stress which contributes significantly to cardiovascular pathology [23,24]. This can explain the phenomenon that an increased consumption of cruciferous vegetables has been negatively correlated with a low risk of cardiovascular disease mortality in a clinic [22,25]. SFN increases the phosphorylation at Ser 1177 of eNOS and NO production in EA.hy926 cells in a time- and dose-dependent manner, as shown in Figure 3, while a decrease in NO production was observed in the presence of L-NAME in Figure 2. In line with the results in recent reports, SFN-rich broccoli sprout extract has a protective role against vascular injury by upregulating the mRNA expression levels of eNOS in HUVECs [26].

Akt activation plays an important role in eNOS phosphorylation at Ser 1177 [27]. Our results clearly showed SFN-induced Akt activation by phosphorylation at Tyr315. PI3K is responsible for Akt activation and the activation of PI3K results in increased eNOS activity and NO production [12,27]. SFN exposure increases PI3K phosphorylation, accompanied by the augmentation of eNOS-Ser 1177 phosphorylation and NO production, suggesting that PI3K is an upstream effector of Akt. An Src tyrosine kinase that frequently serves as a further upstream stimulator of PI3K has been reported to stimulate eNOS phosphorylation through multiple effects [5,27]. We found that SFN increased the phosphorylation of Src in a time- and dose-dependent manner, which was significantly abrogated in the presence of the Src kinase inhibitor PP2, indicating Src as an upstream regulator. The observed results are in line with previous findings that tyrosine kinase Src activation causes eNOS phosphorylation at Ser1177 when exposed to various stimuli [14,27].

## 4. Materials and Methods

### 4.1. Chemicals, Reagents and Antibodies

Sulforaphane (≥95% HPLC), hemin, actinomycin, angiotensin II, N-acetylcysteine (NAC), dimethyl sulfoxide (DMSO), 3-[4,5-dimethylthiazol-2-yl]-2,5-diphenyltetrazolium bromide (MTT) and β-actin were purchased from Sigma-Aldrich Co. (St. Louis, MO, USA). NG-nitro-L-arginine methyl ester (L-NAME), LY294002 (PI3K inhibitor), pyrazolopyrimidines (PP2, an Src kinase inhibitor) were bought from Calbiochem (La Jolla, CA, USA). Primary antibodies for eNOS (32027S), phospho-eNOS (Ser 1177) (9570S), Akt (4691s), phospho-Akt (Ser 473) (4060S), and phospho-Src (Tyr 416), (6943S) as well as horseradish peroxidase-conjugated anti-mouse (7076S) or anti-rabbit IgG (7074S) antibodies were obtained from Cell Signaling Technology (Beverly, MA). All other chemicals, unless otherwise noted, were pure, analytical grade. The cell culture media and reagents involved were obtained from Invitrogen (Carlsbad, CA, USA).

### 4.2. Cell Culture

Human endothelial EA.hy926 cells obtained from the American Type Culture Collection (Manassas, VA, USA) were cultured in a humidified atmosphere containing 5% CO_2_ in an incubator at 37 °C using DMEM medium supplemented with 10% fetal bovine serum (FBS), 100 IU/mL penicillin and 100 mg/mL streptomycin. The chemicals dissolved in DMSO were prepared as stock solutions and were added directly to the culture media. DMSO treatment alone was set as a control group, and the final content of the DMSO was <0.1%.

### 4.3. Cell Viability

Cells were seeded into 96-well plates at a density of 5 × 10^3^ cells/well 24 h prior to the experiments, and transferred to DMEM medium containing 1% FBS for the last 24 h. They were then treated with SFN as indicated. The cell viability was determined by the incubation of 10 μL MTT solution (5 mg/mL) for 2 h. The medium containing an excessive MTT solution was completely discarded and washed with PBS twice, and the formazan granules that were produced were dissolved in DMSO, followed by an absorbance measurement at a wavelength of 562 nm and a reference wavelength of 630 nm on a microplate reader (Biotek Inc., Winooski, VT, USA).

### 4.4. Measurement of NO Production

The NO production was measured using NO-specific fluorescent dyes DAF-2 and DAF-2DA (Calbiochem), as previously described [28]. Both can form fluorescent triazolofuorescein derivative in the presence of NO. DAF-2DA, able to permeate the cell membrane, is converted into membrane-impermeable DAF-2 by intracellular esterases, and is often used to detect intracellular NO. Briefly, human endothelial EA.hy926 cells with 95% confluence were cultured in chamber slides (Lab-Tek, Rochester, NY, USA) and were serum-starved overnight. The cells were treated with DAF-2 or DAF-2DA with a final concentration of 1 μM for 30 min at 37 °C, rinsed three times with DMEM, and incubated in the dark, followed by sulforaphane at indicated concentrations with or without L-NAME (100 μM), LY294002 (10 μM), and PP2 (10 μM) that were added before DAF-2DA treatment for 30 min. Fluorescent signals were measured using excitation and emission wavelengths of 495 and 515 nm, respectively, on a Varioskan microplate reader (Thermo Electron Co, Vantaa, Finland).

### 4.5. Western Blot Analysis

Protein extraction and a Western blot analysis were performed as previously reported [29]. Briefly, cells were seeded at a density of 1 × 10^6^ cells/mL overnight and treated with SFN as indicated. The protein was extracted with RIPA buffer containing protease inhibitors (aprotinin, leupeptin, phenylmethanesulfonylfluoride (PMSF), benzamidine, trypsin inhibitor, sodium or thovanadate) and phosphatase inhibitor. A BCA protein assay kit (Thermo Scientific, Rockford, IL, USA) was used to measure the protein concentration. Proteins were separated in 10–15% sodium dodecyl sulfate polyacrylamide gel electrophoresis (SDS-PAGE). After blocking, the membranes were incubated with the indicated primary antibodies (1:1000). The immunoreactive proteins were developed with Immobilon Western Chemiluminescent HRP Substrate (Millipore, Darmstadt, Germany) in Fusion FX (Vilber Lourmat, Eberhardzell, Germany).

### 4.6. Statistics

Data are shown as mean ± SD and represent the mean of at least three independent experiments. Differences between the data sets were determined using the Student’s *t*-test. The differences described as significant in the text correspond to # *p* < 0.05 and ## *p* < 0.01 versus control.

## 5. Conclusions

Taken together, the present study uncovers a novel effect of SFN to promote eNOS phosphorylation, resulting in the increased production of NO in EA.hy926 cells. The observed effect probably occurs via common upstream Src kinase signaling that leads to the activation of PI3K/Akt signaling. These findings provide clear evidence of SFN in the regulation of eNOS activity and NO bioavailability and suggest that it may be a promising therapeutic candidate to prevent endothelial dysfunction, atherosclerosis and other cardiovascular diseases.

## Data Availability

Not applicable.
